# Supplementary material for: Comparative Analyses of Plastomes of Four Anubias (Araceae) Taxa, Tropical Aquatic Plants Endemic to Africa
Source: Genes (Basel). 2022 Nov 5;13(11):2043. doi: 10.3390/genes13112043 (PMC9690376; doi:10.3390/genes13112043)
Supplement: Supplementary file 1 [file genes-13-02043-s001.zip › Table S1. Plastome characteristics in Anubias and other genera in Araceae.pdf]

**Table S1 Plastome characteristics in *Anubias* and other genera in Araceae**

| Accession number | Species | Total | | LSC | | SSC | | IR | | Coding sequence | | Non-coding sequence | |
|---|---|---|---|---|---|---|---|---|---|---|---|---|---|
| | | Length (bp) | GC (%) | Length (bp) | GC (%) | Length (bp) | GC (%) | Length (bp) | GC (%) | Length (bp) | GC (%) | Length (bp) | GC (%) |
| MH743153 | *Zantedeschia elliottiana* | 175,906 | 35.4 | 88,584 | 34.2 | 8,432 | 28.7 | 39,445 | 37.5 | 95,945 | 39.6 | 79,961 | 30.4 |
| MN046884 | *Anubias heterophylla* | 170,037 | 35.1 | 94,214 | 33.2 | 21,987 | 28.2 | 26,918 | 41.4 | 90,167 | 40.0 | 79,870 | 29.6 |
| MT161480 | *Typhonium blumei* | 169,977 | 35.6 | 90,809 | 33.9 | 15,564 | 29.7 | 31,802 | 39.6 | 95,913 | 39.5 | 74,064 | 30.6 |
| **OP279444** | *Anubias barteri* var. *nana* | 169,929 | 35.2 | 94,504 | 33.1 | 21,993 | 28.3 | 26,716 | 41.6 | 90,206 | 40.1 | 79,723 | 29.6 |
| **OP279443** | *Anubias barteri* | 169,910 | 35.2 | 94,485 | 33.1 | 21,993 | 28.3 | 26,716 | 41.6 | 90,206 | 40.1 | 79,704 | 29.6 |
| **MW984413** | *Anubias hastifolia* | 169,841 | 35.1 | 94,288 | 33.1 | 21,985 | 28.2 | 26,784 | 41.6 | 90,191 | 40.0 | 79,650 | 29.6 |
| MN046885 | *Arisaema franchetianum* | 169,443 | 34.9 | 94,702 | 32.8 | 21,955 | 29.0 | 26,393 | 41.1 | 90,210 | 40.1 | 79,233 | 28.9 |
| MN046892 | *Schismatoglottis calyptrata* | 168,944 | 34.7 | 93,956 | 32.6 | 21,820 | 29.0 | 26,584 | 40.6 | 90,185 | 39.9 | 78,759 | 28.6 |
| MT161478 | *Carlephyton glaucophyllum* | 168,217 | 35.9 | 89,254 | 34.3 | 14,338 | 30.7 | 32,312 | 39.1 | 95,934 | 39.5 | 72,283 | 31.0 |
| MN551187 | *Philodendron lanceolatum* | 167,564 | 35.3 | 94,167 | 33.0 | 21,305 | 29.1 | 26,046 | 42.1 | 90,131 | 40.0 | 77,433 | 29.9 |
| MT226775 | *Zamioculcas zamiifolia* | 167,405 | 35.7 | 91,357 | 34.0 | 19,326 | 29.5 | 28,361 | 40.5 | 90,370 | 40.1 | 77,035 | 30.6 |

**(continued)**

| Voucher | Species | Total | | LSC | | SSC | | IR | | Coding sequence | | Non-coding sequence | |
|---|---|---|---|---|---|---|---|---|---|---|---|---|---|
| | | Length (bp) | GC (%) | Length (bp) | GC (%) | Length (bp) | GC (%) | Length (bp) | GC (%) | Length (bp) | GC (%) | Length (bp) | GC (%) |
| MN046895 | *Taccarum caudatum* | 166,868 | 35.5 | 92,531 | 33.7 | 21,603 | 29.5 | 26,367 | 41.2 | 90,106 | 40.0 | 76,762 | 30.2 |
| MN046889 | *Montrichardia arborescens* | 166,632 | 35.6 | 92,088 | 33.8 | 23,122 | 28.4 | 25,711 | 42.2 | 90,250 | 40.0 | 76,382 | 30.5 |
| MN972442 | *Leucocasia gigantea* | 165,906 | 35.7 | 91,711 | 33.8 | 22,995 | 28.6 | 25,600 | 42.3 | 85,565 | 40.4 | 80,341 | 30.8 |
| MW145396 | *Homalomena occulta* | 165,398 | 35.7 | 92,861 | 33.5 | 20,943 | 29.4 | 25,797 | 42.3 | 90,607 | 39.9 | 74,791 | 30.6 |
| MN046881 | *Aglaonema costatum* | 165,344 | 35.9 | 91,011 | 34.1 | 20,849 | 29.2 | 26,742 | 41.6 | 90,352 | 40.0 | 74,992 | 31.0 |
| MN626718 | *Sauromatum giganteum* | 165,289 | 35.6 | 91,747 | 33.6 | 22,550 | 28.8 | 25,496 | 42.2 | 89,584 | 40.0 | 75,705 | 30.4 |
| MN046886 | *Arisarum simorrhinum* | 164,961 | 36.5 | 87,141 | 35.1 | 14,990 | 30.4 | 31,415 | 40.0 | 90,364 | 40.1 | 74,597 | 32.2 |
| MN046882 | *Alocasia navicularis* | 164,934 | 35.9 | 91,693 | 34.0 | 22,709 | 29.2 | 25,266 | 42.5 | 90,182 | 40.1 | 74,752 | 31.0 |
| MN046894 | *Syngonium angustatum* | 164,929 | 35.7 | 90,714 | 33.9 | 21,559 | 29.0 | 26,328 | 41.5 | 90,136 | 40.0 | 74,793 | 30.6 |
| MT819952 | *Pinellia peltata* | 164,923 | 36.5 | 90,089 | 34.5 | 24,871 | 31.8 | 24,982 | 42.4 | 90,597 | 40.3 | 74,326 | 31.9 |
| MN885890 | *Pistia stratiotes* | 164,551 | 36.0 | 90,705 | 34.1 | 21,886 | 29.1 | 25,980 | 42.3 | 90,270 | 40.2 | 74,281 | 30.9 |

**(continued)**

| Voucher | Species | Total | | LSC | | SSC | | IR | | Coding sequence | | Non-coding sequence | |
|---|---|---|---|---|---|---|---|---|---|---|---|---|---|
| | | Length (bp) | GC (%) | Length (bp) | GC (%) | Length (bp) | GC (%) | Length (bp) | GC (%) | Length (bp) | GC (%) | Length (bp) | GC (%) |
| MT161482 | *Xanthosoma helleborifolium* | 164,418 | 35.8 | 90,833 | 33.9 | 20,705 | 29.9 | 26,440 | 41.5 | 90,384 | 40.0 | 74,034 | 30.8 |
| KR262889 | *Dieffenbachia seguine* | 163,699 | 36.4 | 90,780 | 34.7 | 22,449 | 29.3 | 25,235 | 42.7 | 90,964 | 40.2 | 72,735 | 31.6 |
| MN972441 | *Caladium bicolor* | 163,149 | 35.8 | 89,384 | 34.1 | 21,211 | 29.0 | 26,277 | 41.5 | 88,657 | 40.2 | 74,492 | 30.6 |
| MN046887 | *Calla palustris* | 162,794 | 36.0 | 90,366 | 34.0 | 21,048 | 29.3 | 25,690 | 42.1 | 90,264 | 39.9 | 72,530 | 31.0 |
| MT161483 | *Zomicarpella amazonica* | 162,729 | 35.8 | 90,811 | 34.3 | 21,656 | 28.8 | 25,131 | 41.5 | 89,385 | 39.9 | 73,344 | 30.8 |
| JN105690 | *Colocasia esculenta* | 162,546 | 36.1 | 90,809 | 33.9 | 15,564 | 29.7 | 31,802 | 39.6 | 90,930 | 40.1 | 71,616 | 31.2 |
| MT161479 | *Steudnera colocasiifolia* | 162,500 | 36.1 | 89,806 | 34.4 | 22,196 | 28.9 | 25,249 | 42.5 | 90,308 | 40.0 | 72,192 | 31.3 |
| MK611803 | *Amorphophallus konjac* | 161,647 | 36.4 | 90,006 | 34.3 | 20,197 | 30.7 | 25,722 | 42.3 | 91,143 | 40.1 | 70,504 | 31.6 |
| MN551188 | *Anchomanes hookeri* | 158,177 | 37.0 | 75,594 | 36.0 | 12,903 | 32.3 | 34,840 | 38.8 | 96,189 | 39.3 | 61,988 | 33.3 |
