# Supplementary material for: Comparative Analyses of Plastomes of Four Anubias (Araceae) Taxa, Tropical Aquatic Plants Endemic to Africa
Source: Genes (Basel). 2022 Nov 5;13(11):2043. doi: 10.3390/genes13112043 (PMC9690376; doi:10.3390/genes13112043)
Supplement: Supplementary file 1 [file genes-13-02043-s001.zip › Table S2. 75 protein-coding genes used to construct a ML phylogenetic tree.pdf]

Table S2 75 protein-coding genes used to construct a ML phylogenetic tree

protein-coding genes							
accD	*ndhA*	*ndhK*	*psaI*	*psbL*	*rpl20*	*rps2*	*rps19*
atpA	*ndhB*	*petA*	*psaJ*	*psbM*	*rpl22*	*rps3*	*ycf1*
atpB	*ndhC*	*petB*	*psbB*	*psbN*	*rpl23*	*rps4*	*ycf2*
atpE	*ndhD*	*petD*	*psbC*	*psbT*	*rpl32*	*rps7*	*ycf3*
atpF	*ndhE*	*petG*	*psbD*	*psbZ*	*rpl33*	*rps8*	*ycf4*
atpH	*ndhF*	*petL*	*psbE*	*psbZ*	*rpl36*	*rps11*	
atpI	*ndhG*	*petN*	*psbF*	*rbcL*	*rpoA*	*rps12*	
ccsA	*ndhH*	*psaA*	*psbI*	*rpl2*	*rpoB*	*rps14*	
cemA	*ndhI*	*psaB*	*psbJ*	*rpl14*	*rpoC1*	*rps15*	
clpP	*ndhJ*	*psaC*	*psbK*	*rpl16*	*rpoC2*	*rps18*	
