# Supplementary material for: Comparative Analyses of Plastomes of Four Anubias (Araceae) Taxa, Tropical Aquatic Plants Endemic to Africa
Source: Genes (Basel). 2022 Nov 5;13(11):2043. doi: 10.3390/genes13112043 (PMC9690376; doi:10.3390/genes13112043)
Supplement: Supplementary file 1 [file genes-13-02043-s001.zip › Table S7. Positive selection sites in four Anubias plastomes.pdf]

Table S7 Positive selection sites in four *Anubias* plastomes

Gene name	model	np	LnL	ω8（M8）	LRT P value	Positive sites
ccsA	M8	11	-1312.463533	20.82703	0.007816534	90 Y 0.935,91 F 0.843,92 R 0.636,198 H 0.937,211 Y 0.598,216 L 0.625
	M7	9	-1317.315047			
matK	M8	11	-2062.621266	21.22375	0.014354081	37 L 0.630,46 E 0.618,94 F 0.607,95 D 0.933,214 R 0.660,393 P 0.615,459 P 0.936
	M7	9	-2066.864987			
ndhF	M8	11	-2936.541695	58.20628	0.041312300	49 N 0.579,53 V 0.566,291 M 0.568,463 Q 0.575,571 D 0.568,644 G 0.871
	M7	9	-2939.728290			
ycf4	M8	11	-750.555484	137.62837	0.022086405	129 G 0.886
	M7	9	-754.368277			
