# Supplementary figures and images for: Comparative Analyses of Plastomes of Four Anubias (Araceae) Taxa, Tropical Aquatic Plants Endemic to Africa
Source: Genes (Basel). 2022 Nov 5;13(11):2043. doi: 10.3390/genes13112043 (PMC9690376; doi:10.3390/genes13112043)

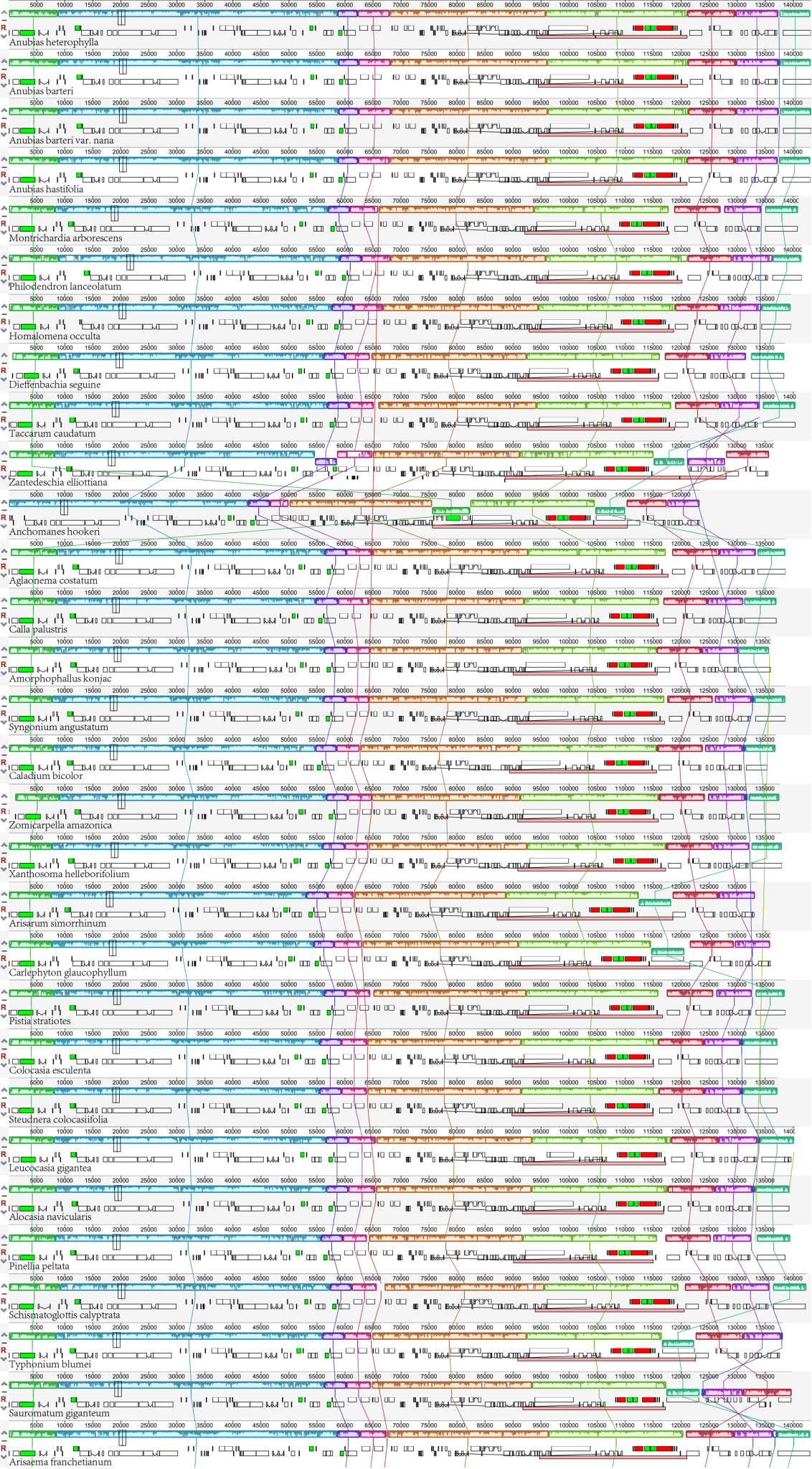

Supplement: Supplementary file 1 [file genes-13-02043-s001.zip › Figure S1. Gene rearrangement analyses among Anubias and other genera in Araceae by Mauve alignment.png]

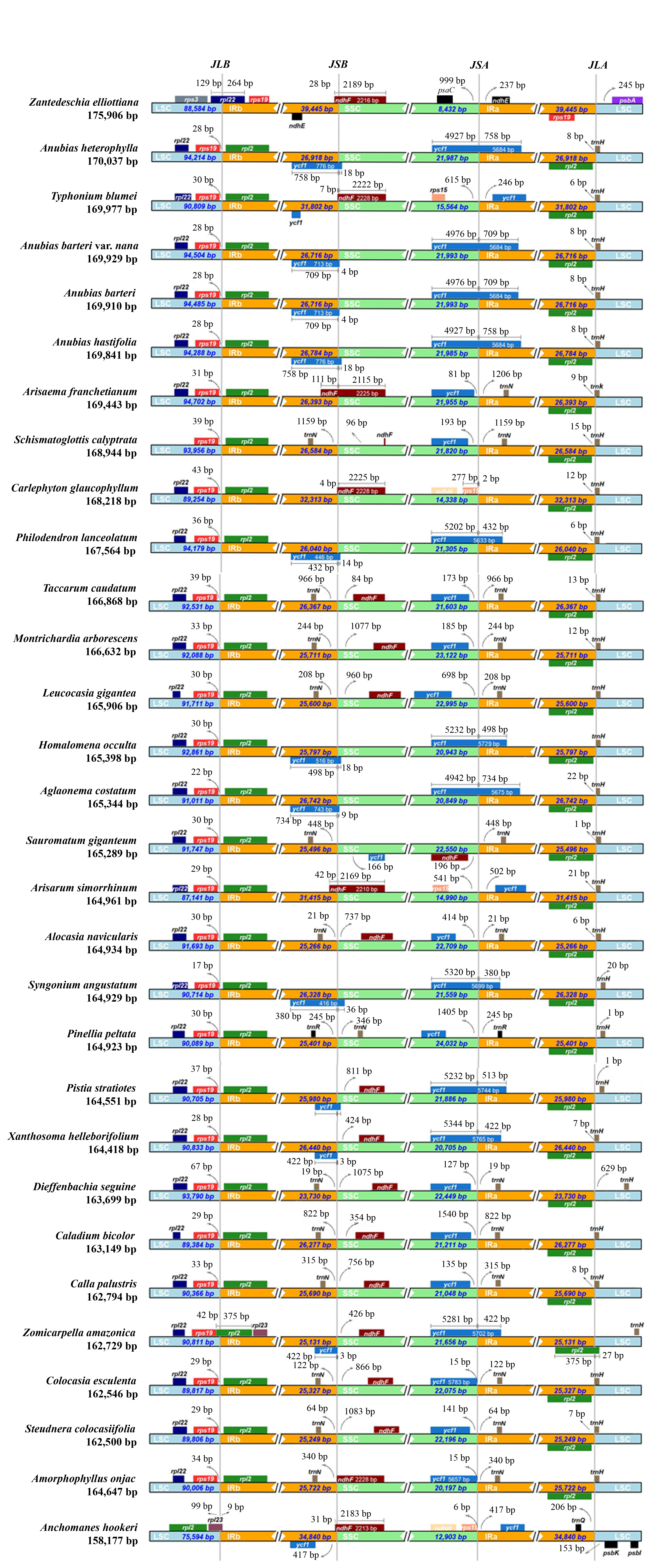

Supplement: Supplementary file 1 [file genes-13-02043-s001.zip › Figure S2. Contraction and expansion of inverted repeats of Anubias and other genera in Araceae.png]
